# Polyurethane Hybrid Composites Reinforced with Lavender Residue Functionalized with Kaolinite and Hydroxyapatite

**DOI:** 10.3390/ma14020415

**Published:** 2021-01-15

**Authors:** Sylwia Członka, Agnė Kairytė, Karolina Miedzińska, Anna Strąkowska

**Affiliations:** 1 Institute of Polymer & Dye Technology, Lodz University of Technology, 90-924 Lodz, Poland; karolina.miedzinska@dokt.p.lodz.pl (K.M.); anna.strakowska@p.lodz.pl (A.S.); 2 Laboratory of Thermal Insulating Materials and Acoustics, Institute of Building Materials, Faculty of Civil Engineering, Vilnius Gediminas Technical University, Linkmenu st. 28, LT-08217 Vilnius, Lithuania; agne.kairyte@vgtu.lt

**Keywords:** polyurethane composites, lavender, kaolinite, hydroxyapatite, high-ball milling process, thermal conductivity

## Abstract

Polyurethane (PUR) composites were modified with 2 wt.% of lavender fillers functionalized with kaolinite (K) and hydroxyapatite (HA). The impact of lavender fillers on selected properties of PUR composites, such as rheological properties (dynamic viscosity, foaming behavior), mechanical properties (compressive strength, flexural strength, impact strength), insulation properties (thermal conductivity), thermal characteristic (temperature of thermal decomposition stages), flame retardancy (e.g., ignition time, limiting oxygen index, heat peak release) and performance properties (water uptake, contact angle) was investigated. Among all modified types of PUR composites, the greatest improvement was observed for PUR composites filled with lavender fillers functionalized with kaolinite and hydroxyapatite. For example, on the addition of functionalized lavender fillers, the compressive strength was enhanced by ~16–18%, flexural strength by ~9–12%, and impact strength by ~7%. Due to the functionalization of lavender filler with thermally stable flame retardant compounds, such modified PUR composites were characterized by higher temperatures of thermal decomposition. Most importantly, PUR composites filled with flame retardant compounds exhibited improved flame resistance characteristics—in both cases, the value of peak heat release was reduced by ~50%, while the value of total smoke release was reduced by ~30%.

## 1. Introduction

Polyurethanes (PUR) are dynamically developing groups of polymers [1,2,3]. Polyurethanes can be used in a variety of applications thanks to the ability to control their mechanical, physical, and chemical properties. Due to this, polyurethane materials are used in many industries, for instance automotive, building and construction, furniture, or industrial insulation [4]. Polyurethanes are obtained by polyaddition reaction between polyols and polyisocyanates, during which the urethane bond, which is the backbone of the resulting polymer, is formed. The first polyurethane was synthesized by Wurtz in 1849, then Otto Bayer in 1937 obtained PUR in the reaction between polyol and polyisocyanate known to this day [5]. The reaction can be carried out in the presence of catalysts, chain extenders, and other additives such as blowing agents or flame retardants [6]. Due to the large selection of substrates used in the reaction, their various mutual ratios, and additives used in the production of PUR materials, it is possible to design the products for a specific application. This allows PUR materials to exist in such forms as foams (rigid and flexible), adhesives, coating, films, sealants, and fibers [7,8]. Among different types of PUR materials, the most commonly used are PUR foams (PUFs), which dominated the market, reaching about 67% of the world’s production of PUR [4].

Rigid polyurethane foams are strongly cross-linked materials. They are characterized by the values of the thermal conductivity coefficient in the range from 0.018 to 0.025 Wm^−1^ K^−1^, which are lower than the values of this parameter, which are achieved by other thermal insulation materials. Due to their thermal insulation and mechanical properties rigid PUR foams are used as low-cost products that find varied applications across many areas such as building insulation, packaging, furniture, transportation, and automotive [4,9,10].

Increasingly stringent environmental requirements for polymeric materials have led to the development of research to improve their mechanical and physical properties by adding ingredients of natural origin [11]. Due to the low price and ecological aspects, special attention is paid to materials of wood and plant origin [12]. One of the methods of modification of rigid PUR foams is the use of bio-fillers which affect their environmentally friendly character. In recent years, there has been a growing interest in the application as natural fillers of raw materials being waste biomass from food processing, including walnut [13] or hazelnut [14] shells and cinnamon [15], coffee, or cocoa [16] extracts. The applied bio-fillers can improve the compressive strength, dimensional stability in critical conditions [17], and thermal insulation capabilities [18] of modified products. It can also change the apparent density and cell structure of modified polyurethane foams and reduce their water absorption [19]. An interesting bio-filler seems to be the distilled lavender residue, which remains after the production of essential oils. Lavender residue is generally considered as waste materials and has been used for composting or burnt to generate energy. An alternative to the traditional uses of solid lavender waste could be its application as a bio-filler in the process of obtaining rigid polyurethane foams. This will allow for the management of the generated waste in a different way [20]. The chemical composition of lavender includes lignin, hemicellulose, and cellulose, significantly affect the properties of the obtained polymer products [21]. As described in the literature, extract of distilled lavender contains interesting volatile molecules including terpenoids and terpenes (e.g., geraniol, linalyl acetate, linalool, and borneol), as well as flavonoids (e.g., luteolin and isoquercitrin), nitrogen compounds (e.g., amino acids, chlorophyll derivatives, and alkaloids) and non-volatile phenolic compounds (e.g., caffeic acid and rosmarinic acid) [22]. It was reported that the extract obtained from distilled lavender shows antioxidant and antimicrobial activity [23]. This suggests that lavender distillation residues can be used as additives to obtain products with better mechanical properties, in parallel, with antioxidant and antibacterial properties [24,25,26].

The main disadvantage that limits the use of PUR foams in many engineering applications is their easy ignition and high flame spreadability [27]. It is known that the degradation of the urethane bond of PUR foams starts at 200 °C [28]. However, it is possible to reduce their combustibility by applying various anti-pyrenes, also known as flame retardants [29]. There are known many types of anti-pyrenes, such as melamine compounds [30], bromine compounds [31], phosphorus compounds [32], inorganic salts [33], or expandable graphite [31,34]. Furthermore, inorganic metal hydroxides and oxides are also mentioned, including compounds of aluminum, magnesium, and silicon, which also play an important role as flame retardants [35,36,37,38]. In the past, halogen compounds were often used as anti-pyrenes, but the growing environmental requirements resulted in the limitation of the application of these substances, due to the release of harmful and toxic gases during the combustion process [10,39]. Therefore, in recent years, the attention of researchers has been focused on the analysis and use of halogen-free flame retardants that do not harm the environment.

One of the flame retardant modifiers of obtained products used in this work is kaolin clay (K) described with the chemical composition of Al_2_O_3_⋅2SiO_2_⋅2H_2_O. It has the form of hexagonal platelets and is a two-layer hydrated alumina silicate consisting of chemically bonded layers of hydrous alumina and silica [40]. Kaolin can be used in many industries due to its low thermal conductivity, chemically inert in a wide range of pH, and exquisite covering power when it is applied as a pigment [41]. Kaolinite is mainly used in the ceramic and paper industry; however, there are studies describing its use in polymer materials [42,43,44]. Kaolin clay can be used also as a flame retardant. Ullah et al. [45] have shown that it is a material that creates ceramic like a protective barrier that reduces the heat transfer to the modified products.

Another flame retardant modifier used in this work is hydroxyapatite (Ca_5_(OH)(PO_4_)_3_) (HA) which is a bio-based polycrystalline calcium phosphate with a hexagonal structure. Its chemical composition includes 39 wt.% Ca, 18.5 wt.% P and 3.38 wt.% OH [46]. The anti-flammable properties of hydroxyapatite have been investigated and proven in various polymer matrices. Researchers investigated the mechanical and thermal performances of HA-modified polymers such as polyvinyl alcohol [47], cellulose [48], and polylactide [49]. For example, Akindoyo et al. [50] reported that the incorporation of 10 wt.% HA into PLA increased the mass residue from 0.35% to 6.17% at 750 °C. Composites of poly(butylene succinate-co-lactate) (PBSL), poly(lactic acid) (PLA) and hydroxyapatite (HA) were investigated by Behera et al. [51]. The results showed that HA was uniformly dispersed in polymeric matrix, while the size of PBSL domain decreased after the addition of HA. In another study, Nabipour et al. [46] have formed a coating containing hydroxyapatite to enhance the fire safety feature of flexible PUR foams and have demonstrated their increased fire resistance.

In the present study, the influence of used modifiers on flammability and rigid polyurethane foams properties was assessed. The fillers used were lavender, kaolin modified lavender, and hydroxyapatite modified lavender.

Although many studies have investigated the influence of cellulosic fillers on PUR foams, there is no studies examined the polyurethane composites filled with lavender filler. Keeping in view, the main disadvantage of PUR foams, which is their high flammability, the current study will focus on the physical functionalization of lavender filler with selected natural, flame retardant compounds—kaolinite (K) and hydroxyapatite (HA) using a high-energy ball milling process. Considering the beneficial properties of lavender and natural flame retardants (kaolinite and hydroxyapatite), it is predicted that the PUR composites developed in this study will exhibit the outstanding mechanical and thermal characteristics, extending their application in the building and construction industry. Because of this, the impact of such developed lavender residue on the mechanical, thermal, insulation, and performance properties of PUR composites will be clearly defined. 

## 2. Materials and Methods

### 2.1. Materials

- Polyether polyol with a brand name of Stapanpol PS-2352 was purchased from Stepan Company (Northfield, IL, USA),
- Polymeric diphenylmethane diisocyanate with a brand name of Purocyn B was purchased from Purinova Company (Bydgoszcz, Poland).
- Potassium octoate with a brand name of Kosmos 75 and potassium acetate with a brand name of Kosmos 33 were purchased from Evonik Industry (Essen, Germany),
- Silicone-based surfactant with a brand name of Tegostab B8513 was purchased from Evonik Industry (Essen, Germany),
- Pentane, cyclopentane, sodium hydroxide (pellets, anhydrous), kaolinite (aluminum silicate, powder), hydroxyapatite (nanopowder, <200 nm) were purchased from Sigma-Aldrich Corporation (Saint Louis, MO, USA)
- Lavender residue was obtained from a local company (Lodz, Poland).

### 2.2. Methods and Instruments

Cell size distribution and morphology of analyzed foams were examined on the basis of the cellular structure pictures of foams that were taken using JEOL JSM-5500 LV scanning electron microscopy (JEOL LTD, Akishima, Japan). The apparent density of foams was determined in accordance with the standard ASTM D1622 (equivalent to ISO 845). The compressive strength (σ_10%_) of analyzed foams was determined in accordance with the standard ASTM D1621 (equivalent to ISO 844) using Zwick Z100 Testing Machine (Zwick/Roell Group, Ulm, Germany). Three-point bending test of analyzed foams was examined in accordance with the standard ASTM D7264 (equivalent to ISO 178) using Zwick Z100 Testing Machine (Zwick/Roell Group, Ulm, Germany). The impact examination was carried out in accordance with the standard ASTM D4812. Surface hydrophobicity was determined by contact angle measurements using the sessile drop method using a manual contact angle goniometer with an optical system OS-45D (Oscar, Taiwan). Water absorption of analyzed foams was analyzed in accordance with the standard ASTM D2842 (equivalent to ISO 2896). The thermal stability of analyzed foams was analyzed using a Mettler Toledo thermogravimetric analyzer TGA/DSC1 (Columbus, OH, USA). Antibacterial properties of PUR composites against selected bacteria and fungi (*Escherichia coli, Staphylococcus aureus, Bacillus subtilis, Candida albicans,* and *Aspergillus niger*) were determined according to the National Committee for Clinical Laboratory Standards. Bacteria were cultured on Tryptic Soy Agar (TSA) medium at 30 °C for 48 h, and fungi on Malt Extract Agar (MEA) medium at 25 °C for 5 days. After incubation, the zones of growth inhibition under and around the film strips were determined. The fire behavior of analyzed foams was performed in accordance with the standard ISO 5660 using the cone calorimeter apparatus in S.Z.T.K. ‘TAPS’—Maciej Kowalski Company (Saugus, Poland). 

### 2.3. Filler Functionalization and Production Process of PUR Composites

**Figure 1:**
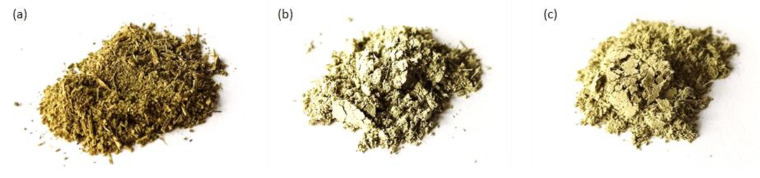
Optical image of (**a**) non-functionalized lavender filler, (**b**) lavender filler functionalized with kaolinite, and (**c**) lavender fillers functionalized with hydroxyapatite.

Before adding to the polyol system, the lavender fillers were alkali-treated, according to the procedure described in [52]. After that, the lavender powder was functionalized with kaolinite (K) and hydroxyapatite (HA). In order to lavender functionalization, a selected amounts of lavender powder and kaolinite/hydroxyapatite (1:1 *w*/*w*) were weighed up and mixed intensively, using a high-energy ball milling process (1 h, 3000 rpm). Such developed lavender fillers (Figure 1) were used as a reinforcing fillers in the synthesis of PUR composites.

**Figure 2:**
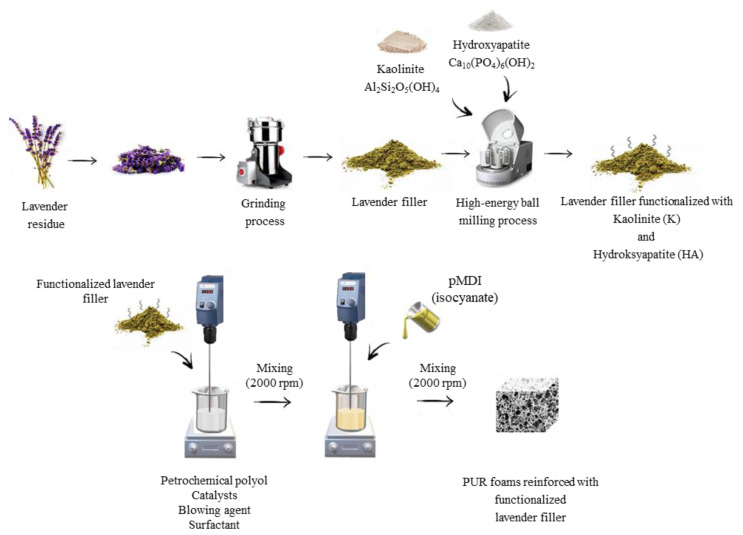
Schematic procedure of the synthesis of polyurethane (PUR) composites filled with non-functionalized and functionalized lavender fillers.

In this regard, the calculated amounts of lavender fillers, polyol, catalysts, surfactant, and blowing agent were placed in a beaker and mixed vigorously (60 s, 2000 rpm). Subsequently, an isocyanate compound was poured into a beaker with vigorous stirring (30 s, 2000 rpm). According to the supplier information, the isocyanate was mixed in the ratio of 100:160 (ratio of polyol to isocyanate) to provide a complete reaction between hydroxyl and isocyanate groups. PUR composites were cured at room temperature for 48 h. The schematic procedure of the synthesis of PUR composites is presented in Figure 2. All formulations of prepared PUR composites are listed in Table 1. 

## 3. Results and Discussion

### 3.1. Filler Characterization

**Figure 3:**
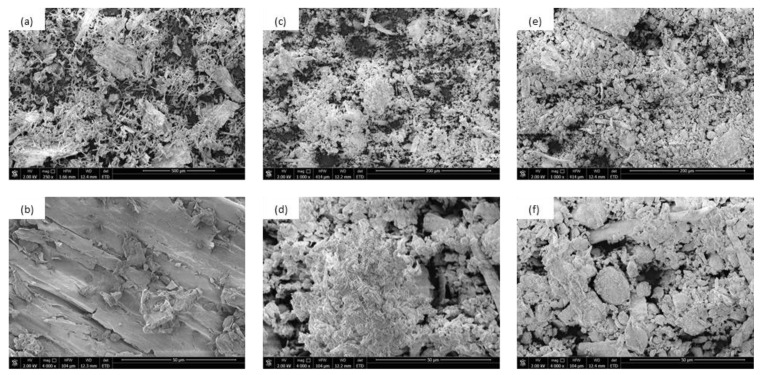
SEM images of lavender fillers: (**a**,**b**) Non-functionalized lavender, (**c**,**d**) lavender functionalized with kaolinite, (**e**,**f**) lavender functionalized with hydroxyapatite.

The functionalization of lavender using a high-energy ball milling process affects the external morphology and size of filler particles. As presented in Figure 3, the external morphology of non-functionalized lavender filler is quite rough, while the size of lavender particles oscillates between 950 nm and 3 µm with an average value at ~1.5 µm. Due to the functionalization of the filler with kaolinite and hydroxyapatite compounds, the overall structure of lavender particles becomes more uniform and smooth, while the average size of filler decreases to 712 and 615 nm, respectively. This may be connected with the fact, that during the continuing ball milling process, the filler particles break into smaller particles, which contribute to the formation of powder fillers with narrow size distribution and a uniform structure. Furthermore, the size of lavender particles affects the viscosity of PUR systems (Table 2). Due to the incorporation of bigger particles of non-functionalized lavender, the viscosity increases from 860 mPa·s (for PUR_REF) to 1215 mPa·s. After the incorporation of lavender filler functionalized with kaolinite and hydroxyapatite, the value of viscosity increases to 1015 and 1050 mPa·s, respectively. 

### 3.2. PUR Composites Characterization

**Figure 4:**
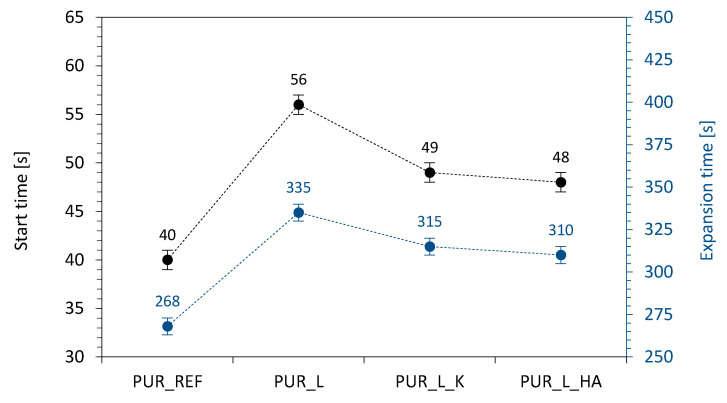
The results of start time and expansion time measured for PUR systems.

As presented in Figure 4, the incorporation of non-functionalized and functionalized lavender fillers affects the processing times in both cases. When compared with PUR_REF, on the addition of non-functionalized, kaolinite-functionalized, and hydroxyapatite-functionalized lavender fillers the start time increases by ~40, ~22, and ~20% while the expansion time increases by ~25, ~17, and ~15%. According to the results presented in previous works, the addition of organic/inorganic fillers may affect the proper stoichiometry of the reaction between isocyanate and hydroxyl groups of polyurethane systems [53]. Due to the presence of filler particles, some highly reactive isocyanate groups react with the active groups introduced by the filler (e.g., hydroxyl groups), limiting the possibility of the reaction between the isocyanate and water and reducing the formation of carbon dioxide (CO_2_). Moreover, the expansion of the cells is additionally reduced by higher viscosity of the modified systems, which explains the extended expansion time of the PUR composites modified with lavender fillers. The analog tendency has been reported in previous studies, which concern the formation of PUR composites modified with another type of organic and inorganic fillers [54].

**Figure 5:**
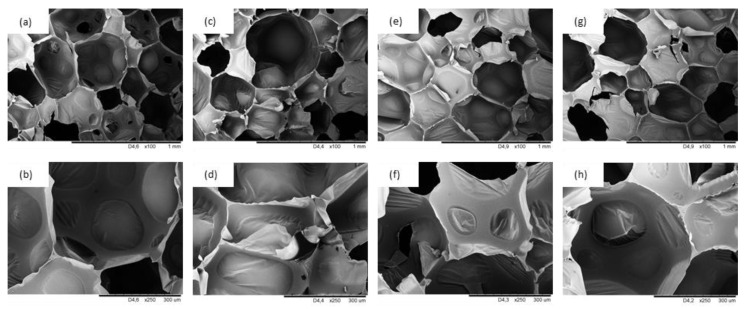
Cellular morphology of (**a**,**b**) PUR_REF, (**c**,**d**) PUR_L, (**e**,**f**) PUR_L_K, (**g**,**h**) PUR_L_HA.

The impact of lavender filler addition on the cellular morphology of PUR composites was evaluated by SEM. As presented in Figure 5a,b, PUR_REF shows the typical, polyhedral structure with a high content of closed-cells. When lavender fillers are added to the PUR systems, an average size of closed-cells tends to be smaller, which is connected with a nucleating effect of the added fillers. The average size of cells decreases from 485 µm (for PUR_REF) to 470, 450, and 455 µm for PUR_L, PUR_L_K, and PUR_L_HA, respectively. According to the SEM results, the incorporation of non-functionalized lavender filler results in the formation of PUR composites with a higher number of open-cells—the content of closed-cells decreases from 87.2 to 85.4% (Figure 5c,d). This may be connected with poor compatibility between the surface of filler particles and the PUR matrix, which results in rupturing and collapsing of the PUR structure and opening of the cells, which in turn, weakens the final structure of PUR composites. The addition of lavender fillers functionalized with kaolinite and hydroxyapatite compounds results in the production of PUR composites with a little more regular structure when compared with PUR composites filled with non-functionalized lavender filler (Figure 5e–h). When compared with PUR_REF, the content of closed-cells increases from 87.2% to 87.9 and 88.2% for PUR_L_K and PUR_L_HA, respectively. This result indicates that the functionalization of lavender filler using a high-energy ball milling process promotes the formation of PUR composites with a higher-crosslinking degree, which prevents the deterioration of the structure during the foaming process. This is additionally enhanced by the presence of solid particles of functionalized lavender fillers which are successfully build into the PUR structure. As discussed previously, the application of a high-energy ball milling process leads to the fracturing of the filler particles, which results in the formation of powder filler with a reduced size of the particles. Due to this, the particles easily build into the PUR matrix, forming new edges for blowing agent encapsulation and enhancing the stability of the overall cellular morphology of PUR composites. Such effect is disturbed in the case of PUR composites filled with non-functionalized lavender fillers, due to the larger size of filler particles. Previous studies have shown, that the application of the filler particles with larger diameters, results in rupturing of the cells, due to incomplete incorporation of the filler particles into the PUR matrix [55]. A similar explanation may be found in our study as well. 

**Figure 6:**
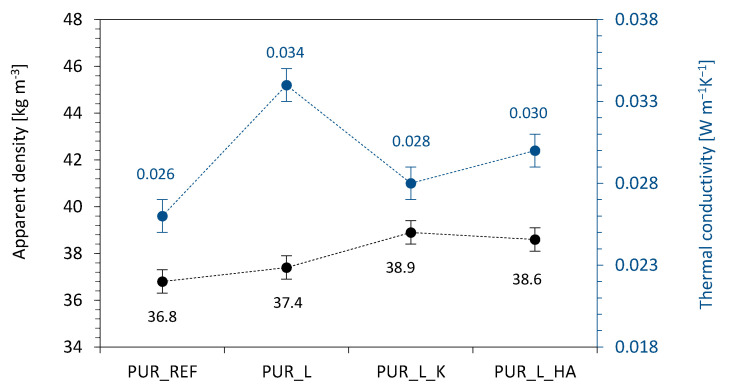
The results of apparent density and thermal conductivity measured for PUR composites.

As presented in Figure 6, on the addition of lavender fillers, the value of apparent density increases from 36.8 kg m^−3^ (for PUR_REF) to 37.4, 38.9, and 38.6 kg m^−3^, for PUR_L, PUR_L_K, and PUR_L_HA, respectively. This may be explained by the fact, that the incorporation of lavender fillers, which are characterized by greater density than the PUR matrix. On the other hand, as shown in Table 2, the incorporation of lavender fillers increased the viscosity of PUR systems, limiting the expansion of the PUR systems and resulting in the formation of smaller cells. Therefore, the density of PUR composites is enhanced.

According to the results given in Figure 6, on the incorporation of non-functionalized lavender filler the value of thermal conductivity (λ) increases from 0.026 Wm^−1^ K^−1^ (for PUR_REF) to 0.034 Wm^−1^ K^−1^, while after the incorporation of functionalized lavender fillers, the value of λ increases insignificantly to 0.028 and 0.030 Wm^−1^ K^−1^. Such an insignificant increase of λ may be attributed to the incorporation of particles of lavender fillers, which increases the heat transfer through the solid particles. Moreover, the most noticeable increase in λ is observed for PUR composites filled with non-functionalized lavender filler. As discussed previously, comparing to PUR_REF, those samples, are characterized by a higher number of open cells. Due to the diffusion through the PUR structure, the gas inside the PUR cells changes from CO_2_ (0.014 Wm^−1^ K^−1^) to atmospheric air (0.025 Wm^−1^ K^−1^), increasing the overall value of λ. A similar trend was observed in previous studies as well [56,57]. For example, Paciorek-Sadowska et al. [58] reported that the addition of rapeseed cake filler in the amount of 30–60 wt.% did not affect the thermal insulation properties of PUR composites—on the addition of 60 wt.% of rapeseed cake filler, the value of λ increased insignificantly from 0.0341 to 0.0348 Wm^−1^ K^−1^.

**Figure 7:**
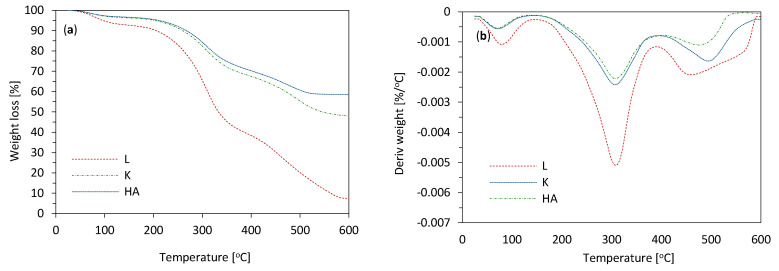

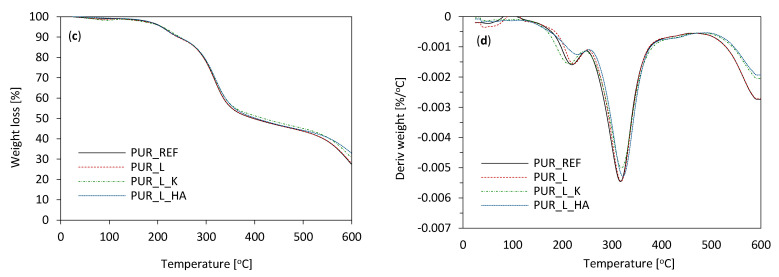
Thermogravimetric (TGA) and derivative thermogravimetry (DTG) results obtained for (**a**,**b**) lavender fillers, and (**c**,**d**) PUR composites.

Thermal stability of lavender fillers and PUR composites was examined using thermogravimetric (TGA) and derivative thermogravimetry (DTG) analysis. The obtained results are presented in Figure 7 and Table 3.

In the case of non-functionalized lavender filler, three stages of thermal decompositions are observed. The first stage of mass loss occurs at relatively low temperature (~100 °C) and refers to the evaporation of the moisture absorbed by the filler and volatile compounds (low molecular weight esters and fatty acids) which are inherent to the filler [59]. The second stage representing 30% of mass loss occurs between 300 and 500 °C. The maximum rate at ~313 °C refers to the thermal degradation of cellulose and hemicellulose [60,61]. The last step of thermal decomposition, representing 42% of mass loss occurs between 400 and 500 °C with the maximum rate at ~460 °C and refers to the thermal decomposition of lignin [61]. When comparing with non-functionalized lavender, the lavender fillers functionalized with kaolinite and hydroxyapatite also revealed three main stages of thermal decomposition; however, the maximum rate of hemicellulose and lignin decomposition is displaced to higher temperatures. This behavior may be connected with the presence of thermal protective layers created by kaolinite and hydroxyapatite, improving the thermal stability of functionalized fillers.

TGA and DTG results of PUR composites are presented in Figure 7. All composites showed three stages of mass loss—T_max1_, T_max2_, and T_max3_. The first stage of mass loss (T_max1_) occurs at relatively low temperatures, between 200 and 250 °C and refers to the thermal decomposition of low molecular weight compounds, which are inherent in lavender fillers [62]. The incorporation of non-functionalized and functionalized lavender fillers results in higher values of T_max1_, indicating the partial crosslinking between lavender fillers and isocyanate groups. T_max2_ occurs between 300 and 350 °C and corresponds to the thermal degradation of hard segments of polyurethane structure and thermal decomposition of lavender fillers [63,64]. Due to the addition of lavender fillers, the maximum temperature of thermal decomposition is displaced to higher temperatures. The greatest improvement is observed for functionalized lavender fillers—the value for T_max2_ increases from 322 °C to 334 and 335 °C, for PUR_L_K and PUR_L_HA. Previous studies have shown, that the addition of filler particles may act as a barrier for heat transfer, effectively inhibiting the further degradation of composites [62]. T_max3_ occurs between 500 and 600 °C and refers to the thermal degradation of lignocellulosic compounds—cellulose, hemicellulose, and lignin [65,66]. When compared with PUR_REF, on the addition of non-functionalized and functionalized lavender fillers, the value of T_max3_ slightly increases, due to the presence of cellulosic fillers and incomplete miscibility of PUR segments (soft and hard segments) [67]. Furthermore, the improved thermal stability of PUR composites was confirmed by the amount of char residue, measured at 600 °C. When compared with PUR_REF, the value increases from 28% to 30, 33, and 35%, for PUR_L, PUR_L_K, and PUR_L_HA, respectively. It may be concluded that filler particles may act as cross-linker points between PUR chains, reducing the heat transfer through the composite structure. The higher cross-linked structure of PUR composites effectively reduces the amount of volatile compounds, which are releasing during the thermal degradation process. Such an explanation may be found in previous studies, as well [62].

**Figure 8:**
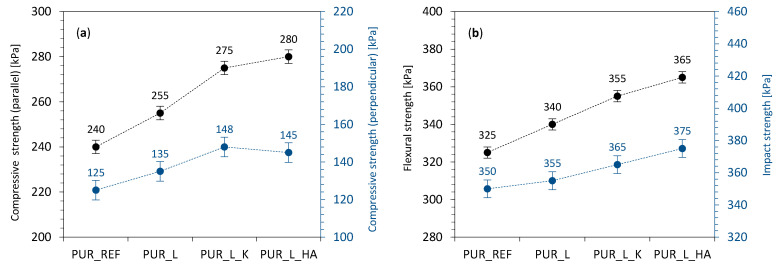
The mechanical performances of PUR foams—(**a**) compressive strength, (**b**) flexural and impact strength.

The impact of lavender fillers addition on mechanical characteristics was evaluated by measuring the compressive strength (labeled as σ_10%_), flexural strength (labeled as σ_f_) and impact strength (labeled as σ_I_). As presented in Figure 8a, the addition of non-functionalized and functionalized lavender fillers affects the value of σ_10%._ When compared with PUR_REF, σ_10%_ (measured parallel to the direction of foam expansion) increases by ~7, ~15, and ~17%, for PUR_L, PUR_L_K, and PUR_L_HA, respectively. An analog trend is observed in the case of σ_10%_ measured perpendicular to the direction of the foam expansion—the value of σ_10%_ increases by ~8, ~18, and ~16% for PUR_L, PUR_L_K and PUR_L_HA, respectively. To avoid the impact of apparent density on mechanical properties of PUR composites, the specific compressive strength was measured as well. Moreover, on the addition of lavender fillers, the specific strength of PUR composites slightly increases—the specific strength (measured parallel) calculated for PUR_REF is 6.5 MPa/kg/m^3^, while due to the incorporation of lavender fillers, the value increases to 6.8, 7.1 and 7.3% for PUR_L, PUR_L_K, and PUR_L_HA. Such improvement may be connected with the morphology features of PUR foams. According to SEM images (see Figure 5), PUR composites reinforced with lavender fillers exhibit a more regular morphology. Thanks to this, the external force is encountered by a greater number of cells which improves the mechanical resistance of PUR composites. The mechanical properties of PUR composites are additionally supported by their more cross-linked structure and the presence of filler particles, which successfully support the load-bearing process [57,68,69]. Most importantly, the obtained results confirm the reinforcing effect of lavender fillers and they are in line with international requirements for constructive materials [70].

A reinforcing effect of lavender fillers was also confirmed by the results of σ_f_ and σ_I_. As presented in Figure 8b, when compared with PUR_REF, the addition of non-functionalized lavender filler increases the value of σ_f_ by ~5%, while the addition of lavender filler functionalized with kaolinite and hydroxyapatite increases the value of σ_f_ by ~9 and ~12%, respectively. A similar trend is observed for σ_I_. The greatest improvement is observed for PUR_L_K and PUR_L_HA—the value of σ_I_ increases by ~4 and ~7%, respectively. As discussed previously, due to the greater number of smaller cells of PUR composite structure, the crack propagation, which is formed under the action of an external load is reduced. Moreover, due to the higher cross-linking degree, the more rigid structure, of PUR composites may absorb more energy, increasing the mechanical resistance of PUR composites [71,72].

In agreement with the literature, lavender residue contains compounds with antibacterial and antioxidative properties, such as phenolic compounds and flavonoids [22]. Antibacterial properties have already been described for kaolinite and hydroxyapatite as well [73,74]. Because of this, the antibacterial properties of PUR composites filled with lavender fillers have been evaluated. The results of the bacterial activity of PUR composites against *Escherichia coli, Staphylococcus aureus, Bacillus subtilis, Candida albicans,* and *Aspergillus niger* are presented in Table 4. The obtained results confirmed the antibacterial activity of PUR composites against bacteria, but no activity against fungi was observed. Low antibacterial activity against fungi, may be connected with low concertation of lavender fillers in the PUR composites. Antibacterial activity of composites filled with lavender has been reported in previous works, however, most studies concern the application of lavender extract. For example, the antimicrobial activity of gelatin-based films containing lavender oils derived from lavender leaves and flowers was investigated by Martucci et al. [26]. Such developed composites exhibited by antibacterial activity against selected bacteria, e.g., *Staphylococcus aureus, Salmonella typhimurium, Escherichia coli,* and *Bacillus subtilis.* Similar results were confirmed in the case of composites filled with lavender oil embedded in sol-gel hybrid matrices [75].

**Figure 9:**
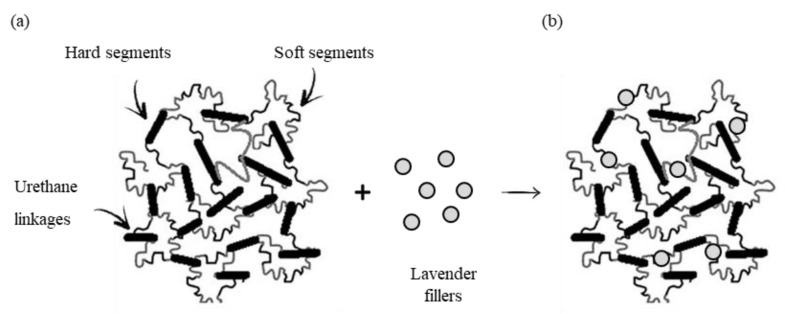
The impact of lavender filler addition on the cross-linking of PUR composites—(**a**) PUR_REF, (**b**) PUR composites with the addition lavender fillers.

**Figure 10:**
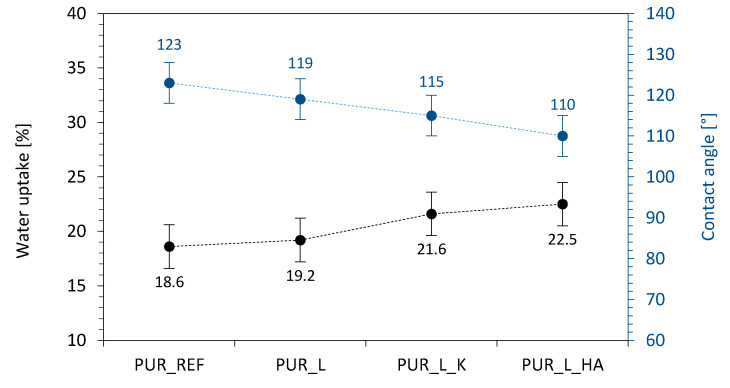
Selected properties of PUR composites—water uptake and contact angle results.

**Figure 11:**
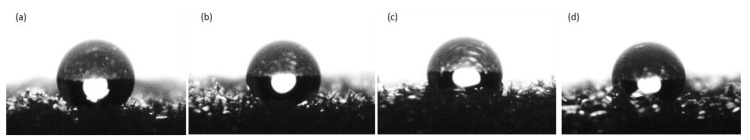
The images of contact angles measured for (**a**) PUR_REF, (**b**) PUR_L, (**c**) PUR_L_K, and (**d**) PUR_L_HA.

Based on the results reported in previous works, the water uptake of porous materials depends not only on the morphology features of porous materials (mostly type of cells—closed or open) but, also depends on the hydrophilic character of the system components, including incorporated fillers [76,77,78,79]. As presented in Figure 9, the water uptake of PUR composites increases by ~3, ~16, and ~21%, for PUR_L, PUR_L_K, and PUR_L_HA, respectively. Based on the SEM results, the PUR composites possess a well-developed structure with a dominant number of closed-cells, which are not able to accommodate the water. Therefore, it seems that an increased water uptake capacity results from the hydrophilic character of incorporated fillers—lavender, as well as lavender functionalized with kaolinite and hydroxyapatite, which also possess a hydrophilic character [80,81,82]. The more hydrophilic character of PUR composites is confirmed by the results of contact angle (Figure 10 and Figure 11)—on the incorporation of lavender fillers, the contact angle decreases from 123° (for PUR_REF) to 119, 115, and 110° for PUR_L. PUR_L_K, and PUR_L_HA, respectively.

The flame retardant abilities of PUR composites were performed using a cone calorimeter. The results of ignition time (IT), peak heat release rate (pHRR), total smoke release (TSR), total heat release (THR), average yield of CO (COY) and CO_2_ (CO_2_Y), and limiting oxygen index (LOI) are presented in Table 5. 

**Figure 12:**
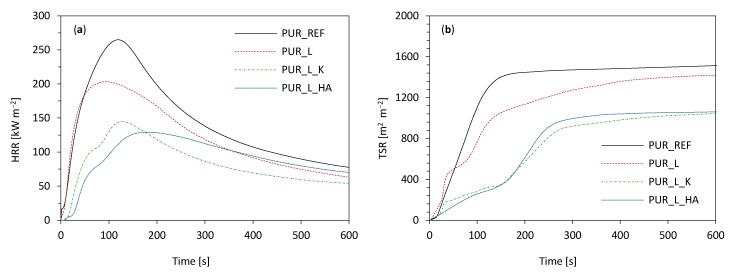
The results of (**a**) peak heat release rate (pHRR) and (**b**) total smoke release (TSR) measured for PUR composites.

Comparing the modified foams to the reference one PUR_REF, it can be noticed that the modifications do not significantly affect the ignition time (IT). As shown in Figure 12a, all series of analyzed PUR composites expose one peak of HRR, which corresponds to the release of low molecular weight compounds, like amines, olefins or isocyanate. Compared to the PUR_REF, which pHRR value is 263 kW m^−2^, modified foams achieve much lower values of this parameter and these the values are respectively 203 kW m^−2^ for PUR_L, 144 kW m^−2^ for PUR_L_K and 130 kW m^−2^ for PUR_L_HA. This may be connected with the formation of a protective char layer, which becomes a physical barrier hindering the flow of heat [83] on the surface of PUR composites. Among all modified series of PUR composites, the lowest value of the pHRR parameter is observed for the PUR_L_HA, which is over 50% lower than for the PUR_REF. As shown in Figure 12b, the incorporation of each filler results in a lower value of total smoke release (TSR). When compared with the PUR_REF, the value of TSR decreases by ~7% for PUR_L, ~29% for PUR_L_K, and ~30% for PUR_L_HA. This suggests that incorporation of lavender fillers protects the PUR structure from further combustion and prevents the heat transfer [84] through the PUR matrix. Furthermore, the incorporation of lavender fillers can decrease the value of total heat release (THR). Comparing to the PUR_REF, for which the value of THR is 21.5 MJ m^−2^, the addition of lavender fillers decreases the value of this parameter to 21.1 MJ m^−2^, 20.5 MJ m^−2^, and 19.8 MJ m^−2^ for PUR_L, PUR_L_K, and PUR_L_HA respectively. As presented in Table 5, the incorporation of lavender fillers decreases the carbon monoxide (CO) to carbon dioxide (CO_2_) ratio, which is related to the foam toxicity. Generally, a higher value of this ratio reveals the incomplete combustion of PUR composites and an increased amount of toxic gases. The application of lavender fillers increases the value of the (CO/CO_2_) ratio, which indicates that the release of toxic gases during the combustion of PUR composites is increased.

As shown in Table 5 the incorporation of lavender fillers effectively increases the value of limiting oxygen index (LOI). The most significant improvement is observed for PUR_L_HA and PUR_L_K—the value of LOI increases from 20.2% (for PUR_REF) to 22.7% for PUR_L_HA and to 22.2% for PUR_L_K. A less noticeable improvement is observed for PUR_L—the value of LOI increases to 20.5%.

**Figure 13:**
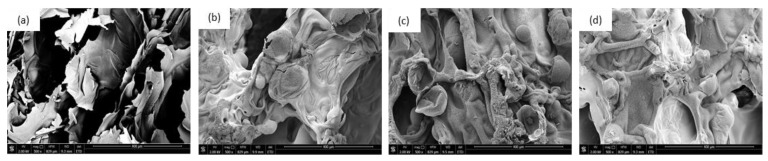
SEM images of char residue of (**a**) PUR_REF, (**b**) PUR_L, (**c**) PUR_L_K, and (**d**) PUR_L_HA (obtained after the cone calorimeter test).

SEM images of char residue of PUR composites after the combustion process is presented in Figure 13. As shown in Figure 13a, after the combustion process, the char residue of PUR_REF seems to be loose and possess a few fragments which were formed during the decomposition process, due to the releasing of the flammable gases. On the other hand, PUR composites filled with lavender fillers, present more compact char residue (Figure 13b–d), which may act as a physical barrier, effectively limiting the heat transfer through the PUR structure. Therefore, the combustion process of PUR composites is successfully inhibiting. 

## 4. Conclusions

Polyurethane (PUR) composites were successfully reinforced with 2 wt.% of lavender fillers functionalized with kaolinite (K) and hydroxyapatite (HA). The impact of lavender fillers on selected properties of PUR composites, such as rheological properties (dynamic viscosity, foaming behavior), mechanical properties (compressive strength, flexural strength, impact strength), insulation properties (thermal conductivity), thermal characteristic (temperature of thermal decomposition stages), flame retardancy (e.g., ignition time, limiting oxygen index) and performance properties (water uptake, contact angle) was investigated. Among all modified types of PUR composites, the best properties exhibited PUR composites filled with lavender fillers functionalized with kaolinite and hydroxyapatite. For example, on the addition of functionalized lavender fillers, the compressive strength was enhanced by ~16–18%, flexural strength by ~9–12%, and impact strength by ~7%. Due to the functionalization of lavender filler with thermally stable flame retardant compounds, such modified PUR composites were characterized by higher temperatures of thermal decomposition. Most importantly, PUR composites filled with flame retardant compounds exhibited improved flame resistance characteristics—when compared with the reference foam, in both cases, the value of peak heat release was reduced by ~50%, while the value of total smoke release was reduced by ~30%. The results reported in the following study confirmed that the application of the high-ball milling process may be an easy and successful attempt in the functionalization of cellulosic filler. The application of such developed fillers in the production of PUR materials is an effective way in the synthesis of PUR composites with enhanced mechanical, thermal, and performance properties.

## Figures and Tables

**Table 1:** Composition of PUR composite foams.

Component	PUR_REF	PUR_L	PUR_L_K	PUR_L_HA
	Parts by Weight (wt.%)			
STEPANPOL PS-2352	100	100	100	100
PUROCYN B	160	160	160	160
Kosmos 75	6	6	6	6
Kosmos 33	0.8	0.8	0.8	0.8
Tegostab B8513	2.5	2.5	2.5	2.5
Water	0.5	0.5	0.5	0.5
Pentane/cyclopentane	11	11	11	11
Lavender non-functionalized	0	2	0	0
Lavender functionalized with Kaolinite (K)	0	0	2	0
Lavender functionalized with Hydroxyapatite (HA)	0	0	0	2

**Table 2:** Rheological and structural properties of PUR composites.

	PUR_REF	PUR_L	PUR_L_K	PUR_L_HA
Dynamic viscosity at 10 rpm (mPa·s)	860 ± 7	1215 ± 9	1015 ± 8	1050 ± 6
Cream time (s)	40 ± 4	56 ± 2	49 ± 2	48 ± 3
Expansion time (s)	268 ± 3	335 ± 8	315 ± 6	310 ± 4
Tack-free time (s)	345 ± 5	330 ± 9	310 ± 7	315 ± 8
Cell size (µm)	485 ± 6	470 ± 5	450 ± 6	455 ± 7
Closed-cell content (%)	87.2 ± 0.7	85.4 ± 0.5	87.9 ± 0.6	88.2 ± 0.4
Apparent density (kg m^−3^)	36.8 ± 0.8	37.4 ± 0.7	38.9 ± 0.5	38.6 ± 0.5

**Table 3:** The results of thermal stability of PUR composites.

Sample	T_max_ (°C)			Residue (at 600 °C) (wt.%)
	1st Stage	2nd Stage	3rd Stage	
PUR_REF	220	322	580	28.0
PUR_L	222	325	585	30.0
PUR_L_K	220	334	590	33.1
PUR_L_HA	235	335	592	35.2

**Table 4:** Antibacterial properties of PUR composites against selected bacteria and fungi.

Sample	Bacteria			Fungi	
	*E. coli*	*S. aureus*	*B. subtilis*	*C. albicans*	*A. niger*
PUR_REF	−	−	−	−	−
PUR_L	+	+	+	−	-
PUR_L_K	+	+	+	-	-
PUR_L_HA	+	+	+	-	-

**Table 5:** Flame retardant properties of PUR composites.

Sample	IT (s)	pHRR (kW m^−2^)	TSR (m^2^ m^−2^)	THR (MJ m^−2^)	COY (kg kg^−1^)	CO_2_Y (kg kg^−1^)	COY/CO_2_Y (−)	LOI (%)
PUR_REF	4	263	1500	21.5	0.210	0.240	0.875	20.2
PUR_L	4	203	1400	21.1	0.190	0.225	0.844	20.5
PUR_L_K	6	144	1060	20.5	0.140	0.190	0.736	22.2
PUR_L_HA	6	130	1055	19.8	0.142	0.180	0.788	22.7
